# Noncompaction Cardiomyopathy—History and Current Knowledge for Clinical Practice

**DOI:** 10.3390/jcm10112457

**Published:** 2021-06-01

**Authors:** Birgit J. Gerecke, Rolf Engberding

**Affiliations:** 1 Department of Cardiology and Pneumology, University Medical Center Göttingen, 37075 Göttingen, Germany; 2 Department of Thoracic and Cardiovascular Surgery, University Medical Center Göttingen, 37075 Göttingen, Germany; 3 Internal Medicine & Cardiology, amO MVZ, Academic Hospital Wolfsburg, 38440 Wolfsburg, Germany; rolf.engberding@gmx.de

**Keywords:** noncompaction cardiomyopathy, NCCM-diagnostic-therapy-prognosis, cardiomyopathy classification, LVNC, LVHT, phenotype, congenital heart disease

## Abstract

Noncompaction cardiomyopathy (NCCM) has gained increasing attention over the past twenty years, but in daily clinical practice NCCM is still rarely considered. So far, there are no generally accepted diagnostic criteria and some groups even refuse to acknowledge it as a distinct cardiomyopathy, and grade it as a variant of dilated cardiomyopathy or a morphological trait of different conditions. A wide range of morphological variants have been observed even in healthy persons, suggesting that pathologic remodeling and physiologic adaptation have to be differentiated in cases where this spongy myocardial pattern is encountered. Recent studies have uncovered numerous new pathogenetic and pathophysiologic aspects of this elusive cardiomyopathy, but a current summary and evaluation of clinical patient management are still lacking, especially to avoid mis- and overdiagnosis. Addressing this issue, this article provides an up to date overview of the current knowledge in classification, pathogenesis, pathophysiology, epidemiology, clinical manifestations and diagnostic evaluation, including genetic testing, treatment and prognosis of NCCM.

## 1. Introduction

When the World Health Organization (WHO) published the first definition of cardiomyopathy in 1980, noncompaction cardiomyopathy (NCCM) was not yet known [1]. Historically, cases with an embryonic spongy pattern of the left ventricle (LV) were described in newborns and infants with complex congenital heart disease, especially with aortic and pulmonary valve atresia [2,3,4,5,6]. In the early cases, diagnosis had to rely on autopsy data, whereas later, newer imaging techniques such as angiography have been used.

**Figure 1:**
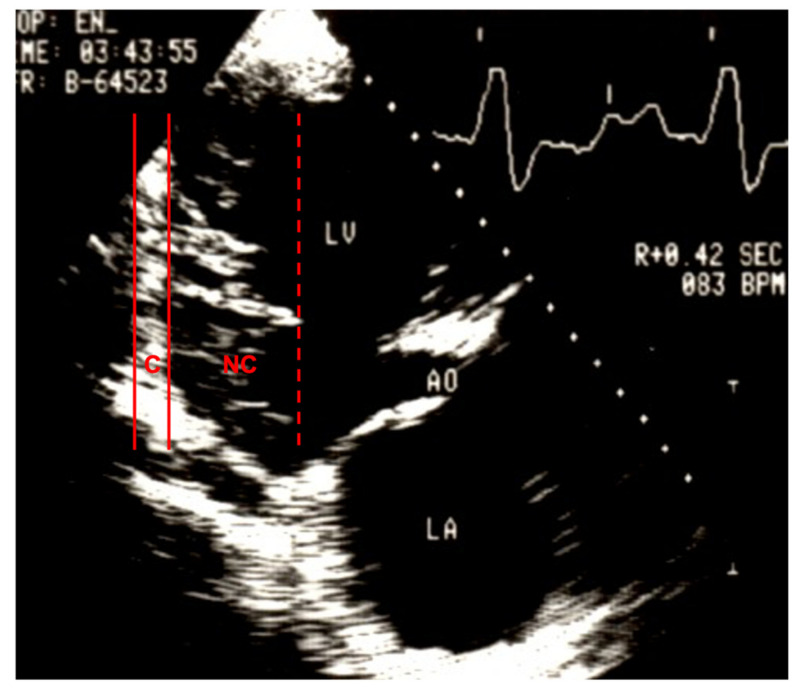
Echocardiography of the first published patient with NCCM without congenital heart disease. LV—left ventricle; LA—left atrium; Ao—aorta; C—compacted layer; NC—noncompacted layer of the left ventricular wall with deep recesses.

A real breakthrough in diagnosis of this myocardial anomaly in vivo could be achieved through 2D echocardiography as was described in 1984 and is presented in Figure 1. The echocardiographic and angiographic images of a 33-year-old female patient showed an embryonic spongy pattern of the LV myocardium in absence of congenital or other structural heart disease [7]. Retrospectively, this was the first published case of NCCM without other structural defects of the heart. In this publication, the myocardial anomaly was referred to as “persistence of isolated myocardial sinusoids”, assuming the anomalous LV morphology was due to a developmental defect in regression of the embryonal sinusoids. This term was used in the following years by other authors for similar cases [8].

In 1990, Chin et al. suggested the term “isolated noncompaction of the left ventricular myocardium (INVM)”, assuming that the myocardial anomaly occurred as a result of an arrest of the normal compaction process during embryonal endomyocardial morpho- genesis [9].

Eventually, recent observations suggest, that LV noncompaction (LVNC) may not be the result of an arrest in the compaction process, but instead results from the compacted myocardium of the ventricular wall, growing into the ventricular lumen in a trabecular fashion [10].

Initially, the term isolated NCCM was used for cases without congenital or other structural heart defects. Today, some groups use the term for cases with areas of LVNC and normal LV function [11]. Table 1 shows a selection of terms and abbreviations used for noncompaction cardiomyopathy and the noncompacted morphology in the last nearly 100 years.

During the years, a wide range of morphological variants could be observed. Some authors, therefore, propose to consider a differentiation in several subtypes, especially in the pediatric population [12].

In the last five years, a number of studies described the usefulness of contemporary diagnostic tools, including echocardiographic imaging techniques, magnetic resonance (CMR), computed tomographic (CT) imaging and genetic testing, and revealed that some cases seemed to occur in patients with neuromuscular disease [13,14,15,16,17]. Towbin and Jefferies discussed NCCM in association with metabolic abnormalities [18].

Of major interest are results from CMR imaging studies, which detected the phenotype of LVNC in healthy athletes and pregnant women, suggesting a physiologic adaptation [19,20]. The same can be found in normal healthy persons, in parts depending on the ethnic group [11]. In this context, future research needs to unravel when the phenotype of LVNC represents pathologic remodeling and when it is a morphologic variant in a healthy person.

## 2. Noncompaction Cardiomyopathy in the Cardiomyopathy Classifications

The classification and definition of cardiomyopathies have been developed in the last six decades with more precise and elaborated recommendations due to increasing abilities in diagnostic and therapeutic modalities.

In 1957, the term cardiomyopathy was proposed for uncommon, noncoronary heart muscle diseases [21]. In 1972, Goodwin and Oakley described cardiomyopathies as myocardial diseases of unknown origin and suggested a first classification [22]. When the first definition and classification of cardiomyopathies by the WHO/ISFC Task Force was reported in 1980, a cardiomyopathy was defined as a heart muscle disease of unknown cause and classified as a dilated, hypertrophic and restrictive cardiomyopathy. “Unclassified cardiomyopathy covered a few cases, which do not fit readily into any group” [1].

The WHO/ISFC updated its classification in 1995 as diseases of the myocardium associated with myocardial dysfunction and now included arrhythmogenic right ventricular cardiomyopathy. The cardiomyopathies were classified by “the dominant pathophysiology or, if possible, by etiological/pathogenetic factors” [23]. Once more the unclassified cardiomyopathies included a few cases that “do not fit readily into any group” (e.g., fibroelastosis, noncompacted myocardium, systolic dysfunction with minimal dilatation, mitochondrial involvement) [23].

In 2006, the AHA defined cardiomyopathies as diseases of the myocardium associated with mechanical and/or electrical dysfunction, which usually exhibit inappropriate ventricular hypertrophy or dilation due to a variety of causes that frequently are genetic, classified as primary or secondary. This classification presented the first attempt to classify primary cardiomyopathy by origin (genetic, acquired, or mixed) and NCCM was assigned a genetic cardiomyopathy [24]. The 2008 ESC proposal defined a cardiomyopathy as a myocardial disorder in which the heart muscle is structurally and functionally abnormal in the absence of coronary artery disease, hypertension, valvular disease and congenital heart disease sufficient to cause the observed myocardial abnormality. The familial diseases included unclassified forms such as NCCM, Barth syndrome, Lamin A/C, ZASP and a-dystrobrevin [25].

Today, NCCM is classified as a genetic cardiomyopathy by the AHA, while the ESC working group for myocardial and pericardial disease and the WHO classified it a familial/genetic unclassified cardiomyopathy [23,24,25].

In 2013 a nosology for cardiomyopathies was proposed with a descriptive genotype–phenotype system, the MOGE(S) nosology [26]. This classification system embodies the morphofunctional phenotype (M), organ involvement (O), genetic inheritance pattern (G), etiological annotation (E), including the genetic defect or underlying disease/substrate, and the functional status (S) of the disease using the American College of Cardiology (ACC)/American Heart Association (AHA) stage and New York Heart Association (NYHA) functional class. The morphofunctional (M) notation provides a descriptive diagnosis of the phenotype (M_D_: dilated cardiomyopathy; M_H_: hypertrophic cardiomyopathy; M_A_: arrhythmogenic right ventricular (RV) cardiomyopathy; M_R_: restrictive cardiomyopathy; M_LVNC_: Noncompaction Cardiomyopathy) [26]. In the MOGE(s) nosology, LVNC is characterized by excessive trabeculation of the LV in echocardiography and cardiac magnetic resonance imaging. The noncompacted ventricular muscle layer is “substantially thicker than the compact layer”; an exact ratio of the layers is not documented. LVNC can occur as an isolated morphological phenotype in association with LV systolic dysfunction or with LV hypertrophy and with mutations in genes typically causing DCM and HCM. The MOGE(S) system distinguishes LVNC with LV dilation and dysfunction (M_LVNC_._D_) or with LV hypertrophy (M_LVNC_._H_) from pure LVNC (M_LVNC_) [26].

## 3. Subtypes of Noncompaction Cardiomyopathy

LVNC remains a heterogeneous morphological anomaly of the heart with multiple possible concomitant phenotypes. Initially, isolated NCCM was characterized by the absence of an additional structural heart disease, especially congenital heart disease; later this definition was changed to those with normal systolic LV dimensions.

In 2015, LVNC was considered to consist of nine distinct subtypes in the pediatric population, whereas another classification was suggested in 2016 [12,27]. These subtypes are listed in Table 2a,b.

In the literature, the most cohorts were analyzed as a whole, and only a few were divided into subgroups for analysis; for example, the isolated NCCM form that met the echocardiographic criteria, but did not have LV dilatation or hypertrophy, or the subtypes with concomitant dilatation or hypertrophy of the LV. Van Waning et al. described four subtypes in their cohort of 349 patients: Isolated NCCM *n* = 95 (27%); NCCM/HCM *n* = 47 (13%); NCCM/DCM *n* = 195 (56%) and NCCM/HCM/DCM *n* = 12 (3%) [28].

Right ventricular noncompaction cardiomyopathy is a challenging diagnosis due to an increased physiological trabeculation of the right ventricle. Nevertheless, there are some cases reported in the literature [29,30].

## 4. Epidemiology

NCCM in the pediatric and adult population probably has a different background. The National Australian Childhood Cardiomyopathy study documented 9.2% of the primary cardiomyopathies in children younger than 10 years of age as NCCM [31,32]. Jefferies et al. observed in the pediatric cardiomyopathy registry of the National Heart, Lung, and Blood Institute 4.8% of children with NCCM [32]. Therefore, NCCM was the third most common type of cardiomyopathy after DCM and HCM [31,32,33].

The percentage in adults seems lower: 4.1% in the EORP Cardiomyopathy registry and 5% in the German Torch registry [34,35].

In men, NCCM is twice to three times more common than in women. The diagnosis can be achieved in adults, adolescents, children, newborns and even prenatal [36,37]. The prenatal identification of isolated NCCM is feasible with current ultrasonographic technology in the hands of an experienced examiner who is familiar with the features of this rare anomaly. Fetal IVNC can involve the LV, the RV, or both ventricles [38]. Sato described a patient with NCCM in the 10th decade of life presenting with a transient cerebral ischemia [39].

Left ventricular noncompaction is estimated to affect 8 to 12 per one million individuals per year, but the condition is likely more common because asymptomatic individuals are not diagnosed. Increased awareness of this condition and improvements in noninvasive cardiac imaging have led to a higher detection rate.

Familial occurrence is seen in up to 40% of the cases. The clinical phenotype is very variable, even in a single family. In a systematic family screening, a quarter of the examined relatives showed echocardiographic abnormalities, including LV dysfunction with and without noncompaction [40].

In experienced echocardiography departments NCCM has a prevalence of 0.014% to 0.26% [17,41,42,43]. With more sensitive imaging techniques the numbers increased. The prevalence with echocardiographic imaging in cardiac patient cohorts rose to about 0.9%, in healthy controls 1.05%, in athletic cohorts 3.16% and in pregnant cohorts up to 18.6% [44]. With MR imaging the numbers were significantly higher, with 9.6% in cardiac patients and up to 36.2% in subgroups [44]. Cardiac MR imaging in asymptomatic, healthy volunteers met varying noncompaction criteria in 1.3 to 14.8% [45], in other adult populations the numbers differed with the diagnostic criteria applied between 3% and 39% [15]. Thus, the real number seems to be unclear due to selection bias in different cohorts. The significance of the findings in asymptomatic peoples remains unclear.

## 5. Clinical Features

There is a broad spectrum of clinical presentations with primarily heart failure symptoms, different forms of arrhythmias and thromboembolic events. Between the onset of symptoms and the diagnosis there can be a delay of up to 3 to 4 years [43].

Heart failure symptoms can be mild, but severe symptoms with need for heart transplantation or LV assist device implantation can arise. In the German NCCM registry, 61% of patients showed heart failure symptoms at the time of initial diagnosis of NCCM and 15% developed heart failure symptoms during the follow-up of 27 months or deteriorated [46].

Arrhythmias are frequent in NCCM, ventricular as well as supraventricular arrhythmias. In the German NCCM registry 26% of the patients presented with arrhythmias and were subsequently diagnosed with cardiomyopathy. In 17%, atrial fibrillation was found. Atrial fibrillation was more often observed in patients with reduced LV function. Bradycardia requiring pacemaker implantation was seen in 5% of the cohort. Supraventricular arrhythmias occurred in 4%, WPW syndrome was observed in 1.5%, AV nodal reentry tachycardia in 1% and typical atrial flutter in 1.5%. Sustained ventricular tachycardia and ventricular fibrillation were observed in patients with severely reduced LV function. In patients with an LV ejection fraction above 35%, sudden cardiac deaths were found only rarely [47,48]. In children, up to 20% presented with WPW syndrome and with sinus bradycardia; WPW syndrome can be associated with cardiac dysfunction [49]. Table 3 summarizes observed arrhythmias in the German NCCM registry.

Thromboembolic events mainly occur in patients with NCCM and atrial fibrillation. Stasis of blood flow can appear in the deep intertrabecular recesses notably in reduced LV function. Neurologic departments occasionally diagnose NCCM in patients with otherwise not explained stroke. In cohorts with NCCM, a percentage of about 10–15% suffer from stroke [50].

There are several case reports of patients with NCCM and specific neuromuscular disorders or hereditary neuropathy such as Charcot–Marie–Tooth, Becker muscular dystrophy, Emery–Dreifuss muscular dystrophy, myotonic dystrophy, Leber’s hereditary optic neuropathy, and Barth syndrome, and also with neuromuscular problems not specified in more detail [17,51]. These findings support the necessity of systematic neurological examination in patients with NCCM [52].

Several congenital heart defects were described with NCCM [53,54]. A patient with pulmonic valve atresia was described in 1964 and patients with Ebstein anomaly in 2005 [5,55]. Friedberg described a patient with atrial isomerism [56]. Shunt defects such as ventricular septal defect, atrial septal defect and patent ductus arteriosus Botalli were described as well [57]. Stähli described noncompaction in patients with congenital heart disease, in Epstein anomaly, subaortic VSD, bicuspid aortic valve and tetralogy of Fallot [54]. This in fact strengthens the need for a comprehensive echocardiographic evaluation of any patient with newly diagnosed NCCM to rule out congenital heart disease. In about 50% of children with concomitant face dysmorphisms or a neutropenia (Barth syndrome), cardiomyopathy with and without noncompaction was described [58].

Coronary heart disease is uncommon in NCCM, but severe coronary heart disease that needs revascularization therapy has been found in some patients with NCCM [7,59,60,61].

## 6. Diagnostic Criteria

Up to now, the diagnostic criteria of LVNC are far from being perfect [62]. The differential diagnosis between NCCM and normal phenotypic variants cannot be established properly in a lot of cases. Even using multimodality imaging, including echocardiography and cardio MR, the criteria are not specific enough to properly avoid under- or overdiagnosis with important implications on treatment strategies or prognostic estimations. The current available diagnostic imaging criteria show a propensity towards overdiagnosing NCCM [63].

### 6.1. Diagnostic Criteria for Echocardiography

**Figure 2:**
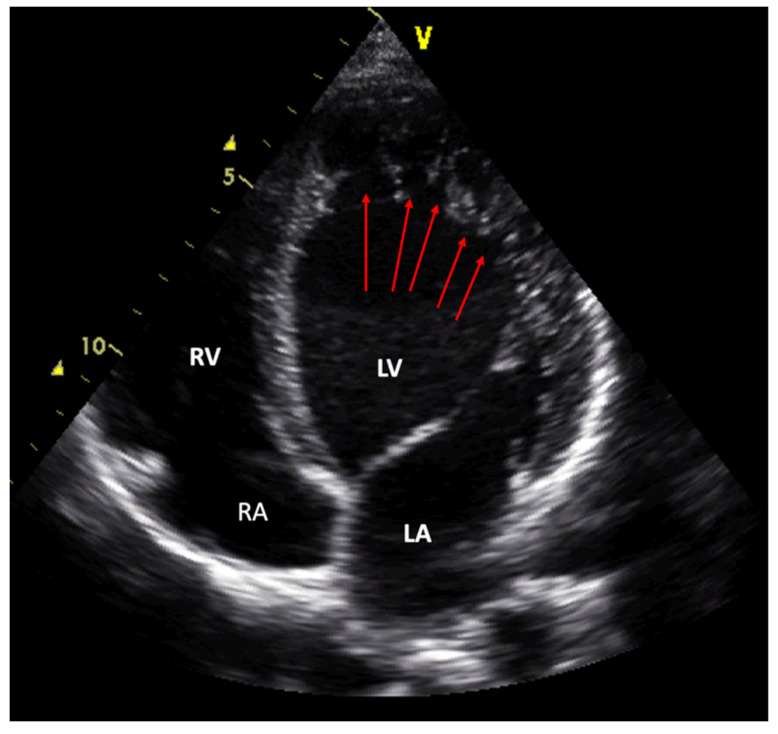
An echocardiographical apical 4-chamber view in a patient with NCCM. LV shows mild dilatation. The arrows mark the deep recesses in the noncompacted layer of the apical and lateral LV wall. LV—left ventricle; LA—left atrium; RV—right ventricle; RA—right atrium.

Echocardiography with its widespread availability, low costs and nearly zero complications is the first-choice procedure in diagnosis of LVNC. Echocardiography can detect the pathognomonic features of a thick, bilayered myocardium with prominent trabeculations and intertrabecular recesses communicating with the LV cavity (Figure 2). If conventional echocardiography is not diagnostic, additional contrast echocardiography is suggested in the EACVI recommendations [64]. Transesophageal echocardiography or real time 3D echocardiography—if applicable, combined—have also been shown to be helpful diagnostic procedures in cases with LVNC [65,66].

LVNC can be mainly observed at the cardiac apex and in the mid-inferior, mid-anterior, and mid-lateral areas of the LV wall [67]. A low-Nyquist limit color mapping is recommended to show blood flow into the recesses.

Although the first echocardiographic diagnosis of NCCM was published more than 37 years ago and the term noncompaction was introduced more than 30 years ago, there are no globally accepted diagnostic criteria [7,9,68]. Chin et al. were the first who introduced the term noncompaction and suggested diagnostic criteria for their cohort of children and young adults (California criteria). Jenni et al. described the criteria in a group of adults (Zurich criteria), both using the ratio between the compacted and the noncompacted layer, but with different measurements and timing in the cardiac cycle [8,9]. Stöllberger in contrast focused on the number of trabeculations and only later used the ratio as an additional diagnostic attribute (Vienna criteria) [17]. The German noncompaction registry used the combination of Jenni and Stöllberger criteria (Table 4.) [62,69]. Each group excluded patients with additional heart disease. In 2012, Paterick altered the measurement interval of the Jenni criteria; thus, creating the Milwaukee criteria [70].

Frischknecht recommended the combination of criteria as specific, and Belanger in 2008 proposed different criteria with an additional classification of the severity derived from the noncompaction/compaction ratio and the affected planimetered area in cm^2^ on an apical 4-chamber view, with three categories: mild, moderate and severe [71,72].

In the last decade of the last century and the first decade of this century, the diagnosis of NCCM was confirmed mainly in heart failure patients, and underdiagnosis of the disease was of major concern. Since more common recognition of the disease and introduction of newer imaging techniques occurred, the problem of overdiagnosis arose. To overcome these problems, additional criteria were introduced by the Swiss group in 2012: the compacta thickness; a compacta thickness of below 8 mm being a discriminator between the groups [73]. Sabatino tried to discriminate between NCCM and LVHT in a pediatric cohort using the noncompacted to compacted end diastolic myocardial ratio: the noncompaction cardiomyopathy with >2.3, the LVHT with <2.3 and >1.7 [74].

The Rotterdam group was the first to show that the absence of twist in speckle tracking tissue Doppler echocardiography can be a marker for NCCM. They demonstrated the loss of LV twist in 83% of 34 adults with NCCM [75]. The radial wall motion and the longitudinal LV wall velocity is impaired in NCCM, but the findings do not correlate with the extent or severity of noncompaction [76]. Further studies also showed impaired LV twist and the presence of rigid body rotation in NCCM but not in LVHT. A correlation between LV twist reduction in apical rotation and LV function was observed [77]. Table 5 shows an overview of different echocardiographic criteria.

Arunamata et al. investigated the speckle tracking strain results in a pediatric study group with NCCM, with and without congenital heart defects. Segmental radial, circumferential, and longitudinal strain decreased in NCCM compared with control subjects. Strain measurements were lowest in those with adverse compared with favorable outcomes. In NCCM, deformation was affected in all regions, including compacted myocardial segments [79].

Sabatino et al. assessed global and regional longitudinal strains in a pediatric population using apical 4-chamber, 3-chamber, and 2-chamber views; radial and circumferential strains were measured using LV short-axis views at different levels. LV twist was calculated as the difference between peak apical rotation and basal rotation at a time interval corresponding to the ejection phase of the systole. The measurements could discriminate between a normal counterclockwise pattern with reduced apical rotation peak values in LVHT and NCCM with rigid body rotation presenting with a sensitivity of 82% and a specificity of 92% [74]. In contrast, Huttin et al. showed that myocardial deformation was preserved in the apical region [80].

The World Heart Federation MOGE(S) classification graded LVNC as a morphological entity with an excessive trabeculation of the LV on echocardiography or cardiac magnetic resonance imaging. The noncompacted ventricular muscle layer was substantially thicker than the compact layer. No ratio was specified [26].

Even in experienced groups the interobserver agreement of the echocardiographic diagnosis was limited, with about 11% of questionable cases [81,82]. Measurements, according to existing diagnostic criteria for NCCM, vary due to the echocardiographic view and segment with different interobserver reliability and predictive validity. In a pediatric population, Joong et al. observed that the NC/C ratio showed the lowest reliability and predictive validity [83]. They found that the end diastolic measurements were more precise than the end systolic. A single echocardiographic diagnostic study may be too sensitive and may lead to overdiagnosis. Kohli et al. reported that 23.6% of patients presenting to their heart failure clinic met at least one of the three echocardiographic criteria for NCCM, including 8.3% of healthy control subjects, 50% of the control persons were black [84].

### 6.2. Diagnostic Criteria for Magnet Resonance Imaging

**Figure 3:**
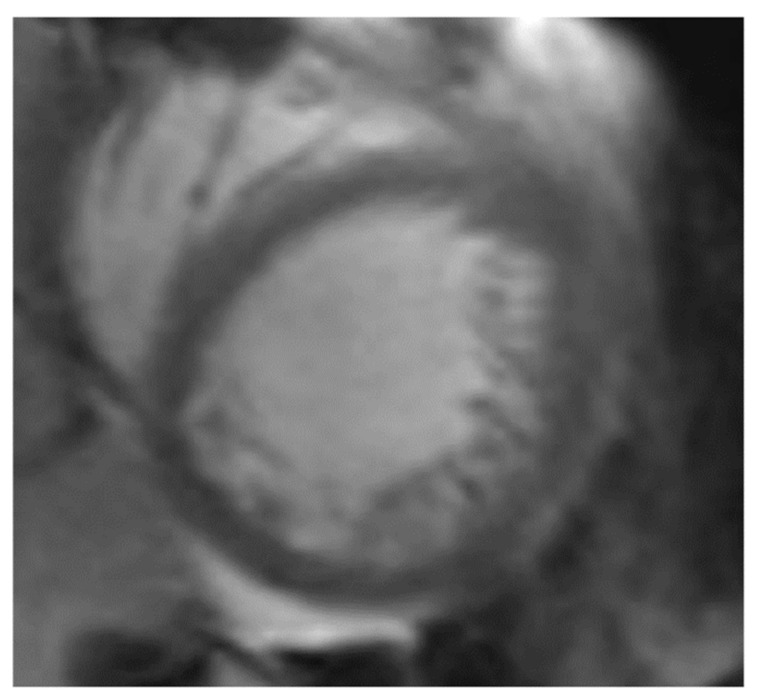
Magnetic resonance imaging: short axis of the left ventricle with excessive trabeculations. Notable, the septum shows no trabeculation.

NCCM can be diagnosed using CMR (Figure 3.) There are several different methods that have been proposed to diagnose NCCM using CMR. Peterson et al. evaluated an end diastolic NC:C Ratio ≥ 2.3 measured in long axis cine views at the site with the most pronounced trabeculations, while Stacey defined an end systolic ratio > 2 in short axis views to diagnose NCCM [85,86]. Jacquier used short axis views to measure trabecular mass, where more than 20% of noncompacted mass were defined as NCCM [87]. Captur et al. described an end-diastolic loss of the base to apex fractal dimension gradient ≥ 1.3 for NCCM patients [88]. Grothoff redefined and extended the MR imaging criteria for diagnosing and discriminating NCCM from other cardiomyopathies using four basic criteria: the percentage of LV noncompacted myocardial mass (positive with >25%), the total amount of LV noncompacted myocardial mass (MM; positive >15 g/m^2^), a noncompacted to compacted myocardium ratio of ≥3:1 in at least one of the segments 1–3 or 7–16 excluding the apical segment 17 and trabeculation in segments 4–6 ≥ 2:1 (noncompacted to compacted ratio) [89]. Dreisbach et al. used strain on MR imaging and Dodd et al. examined trabecular hyperenhancement on cardiac MR imaging [90,91]. The different MR criteria are listed in Table 6.

## 7. Additional Diagnostic Armamentarium

### 7.1. The Multimodality Imaging Approach

A combination of different echocardiographic criteria and, if appropriate, a combination with an additional diagnostic technique should help to diagnose NCCM definitely or reject the diagnosis. However, there may be some borderline cases with a possible, but not definite, diagnosis of NCCM. In these cases, the imaging approach has to integrate clinical findings, family history and genetic data. However, at this time, a negative genetic test is not a marker, that diagnosis of NCCM is unlikely [28,93].

### 7.2. Left Ventricular Angiography

Diagnosis of NCCM can also be performed by LV angiography. Sometimes it is a diagnosis at a glance, but there are no definite criteria for the diagnosis of NCCM by LV angiography [7,62].

### 7.3. Computer Tomography

Cardiac CT is an imaging tool with increasing significance. In patients with dilated cardiomyopathy, cardiac CT is used to exclude coronary heart disease [94]. Conces et al. reported the first diagnosis of NCCM using cardiac CT [95]. For diagnosis of NCCM by cardiac CT, Melendez-Ramirez et al. proposed a ratio of noncompacted to compacted layer of 2.2 in one segment [96]. Fuchs et al. analyzed ECG-triggered low-dose cardiac CT and could discriminate patients with NCCM from normal individuals by using an NC:C ratio of >1.8 in diastole. Their results showed a good correlation of NC:C ratio between transthoracic echocardiography (TTE) and cardiac CT with the threshold of 1.8 [97].

### 7.4. Electrocardiography

An ECG does not show specific alterations in cases with NCCM. Alterations in the ST segments and T waves are common, as also are different types of bundle branch blocks. Some publications reported that nearly 90% of the patients presented with ECG alterations [98]. Conduction delay, P-wave abnormalities, QRS-axis deviation, interventricular conduction defects and various forms of bradyarrhythmias and tachyarrhythmias have been observed in affected patients. Alterations induced by a Wolff–Parkinson–White (WPW) syndrome may especially occur in children [49]. Atrial fibrillation is frequently observed [47,48].

### 7.5. Biomarkers

NTproBNP is a marker for heart failure. High NTproBNP levels were investigated for being an indicator for death and heart transplantation in patients with NCCM [99]. An elevated troponin level can refer to myocarditis but may also be present in patients with NCCM [100].

### 7.6. Endomyocardial Biopsy

Endomyocardial biopsy continues to be the gold standard in the detection of myocardial inflammation. Myocarditis is a potential differential diagnosis in cases with NCCM. Biopsy findings can offer clear therapeutic recommendations, especially in cases with giant cell myocarditis or sarcoidosis.

## 8. Differential Diagnosis

Prominent LV trabeculation can be found in healthy hearts, as well as in hypertrophic cardiomyopathy (HCM) and in LV hypertrophy secondary to dilated, valvular, or hypertensive cardiomyopathy. Thus, the differentiation between variants and LVNC may often be challenging [101]. Differential diagnosis of NCCM includes apical and other located LV thrombus, false tendons, aberrant chords, cardiac fibromas, eosinophilic heart disease, endomyocardial fibrosis and cardiac metastasis [102]. Other cardiomyopathies or localized LV hypertrophy have to be discriminated. Myocarditis may imitate NCCM.

In 25% of normal pregnancies, an increase in LV trabeculations can be assessed [20,103]. A LVNC pattern in pregnancy was reported in several studies [20,104,105]. Diagnosis of NCCM, therefore, is more difficult in pregnant women. A peripartum cardiomyopathy and a preexisting NCCM are important differential diagnoses in pregnant women or women after delivery with heart failure symptoms, requiring different treatment options. Noncompaction of the LV myocardium may be the morphological trait of a physiological remodeling in these cases.

Some persons affected with noncompacted areas do not have any symptoms and are diagnosed by chance. A major number of these persons are athletes, and noncompaction perhaps may be a physiological remodeling process in these persons [19]. Luijkx et al. even found ethnic differences in athletes with a greater degree of LV trabeculation in healthy African athletes, combined with biventricular EF reduction at rest [106]. De la Chica et al. found hypertrabeculation in persons with vigorous physical activity [107]. To exclude NCCM in athletes, a pre-participation screening with clinical and family history, ECG and echocardiography, and, in suspicious findings in these examinations, a CMR was recommended [19].

**Figure 4:**
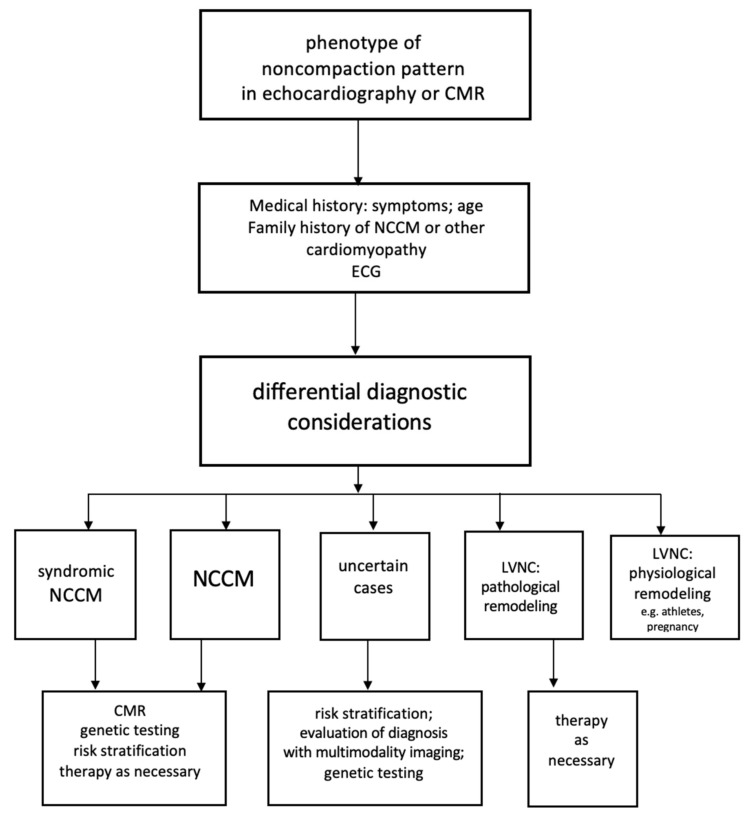
A pathway for differential diagnosis and risk stratification in patients with noncompacted myocardium (LVNC).

A noncompaction pattern can be a myocardial response to acquired triggers, Loria et al. discussed chemotherapy with drug toxicity as a possible trigger [108]. A report of 2009 described diagnosis of LVNC in a group of family members, including a pair of identical twins; each of them suffered from thalassemia major requiring multiple transfusions, and suggested a possible association with cardiac siderosis [109]. Chronic renal failure and polycystic kidney disease were reported likewise [27]. Figure 4 proposes a pathway for differential diagnostic considerations.

## 9. Pathogenesis—Embryogenesis and the Pathophysiological Concept

In the first series of patients with NCCM, the disease was familial to a large extent. Noncompaction areas resembled the fetal heart and the hypothesis of an arrest in the normal compaction process of the heart seemed adequate.

The development of the heart is a complex, precisely regulated molecular and embryogenetic process. The different steps of the development are triggered by specific signaling molecules and mediated by tissue-specific transcription factors [110,111]. Trabeculations appear at the end of the fourth gestational week in humans, when the heart tube consists of an external myocardial layer and an internal endocardial epithelium. The first trabeculations appear in the cardiac jelly between endocardial–myocardial contact points and extend radially into the ventricular lumen [112]. During myocardial development, two different myocardial layers are formed within the ventricular wall, a trabecular layer and a compact subepicardial layer [113,114,115]. In gestational week 12, development of trabeculations increases the surface to enhance the blood, respectively oxygen supply of the growing myocardium prior to the developing of the coronary arteries. The resulting intertrabecular recesses communicate with the LV cavity. The next step in the development is a compaction process from basal and septal to apical and lateral LV areas. This process underlies a complex genetic regulation as well. An arrest of the compaction process can occur if signal molecules are not expressed at the correct time [116].

The NOTCH pathway is required for proliferation, differentiation and tissue patterning in various tissues, including the heart [117]. The NOTCH pathway seems to independently regulate cardiomyocyte proliferation and differentiation, two balanced processes whose perturbation may result in congenital heart disease. Mutations in the NOTCH pathway regulator MIB1 cause NCCM by impaired growths of the trabecular instead of the compacted layer [118]. Other mutations in the NOTCH pathway lead to incorrect marker expression (e.g., EphrinB2, NRG1, BMP10, and MIB1) and decreased myocardial proliferation [112]. Neuregulin, ErbB2, ErbB4 and Nkx2.5 code other signaling proteins regulating organ proliferation and are described to control myocardial cell outgrowth that ultimately results in trabeculation [119,120,121]. The significance of the mutations in the NOTCH pathway was demonstrated by mutations in mice that lead to noncompacted myocardium [118].

In 2016, Jensen in contrary described the excessive trabeculations in noncompaction not to have the embryonic identity and drew the conclusion that noncompaction is probably not the result of failed compaction, but likely the result of abnormal growth of the compact wall [10]. This means that no compaction process may be present in the embryonic endomyocardial morphogenesis and that the term LVHT may be more appropriate than LVNC [68]. However, up to now, the concept of an arrest in the endomyocardial development is not completely understood [117].

## 10. Pathology

**Figure 5:**
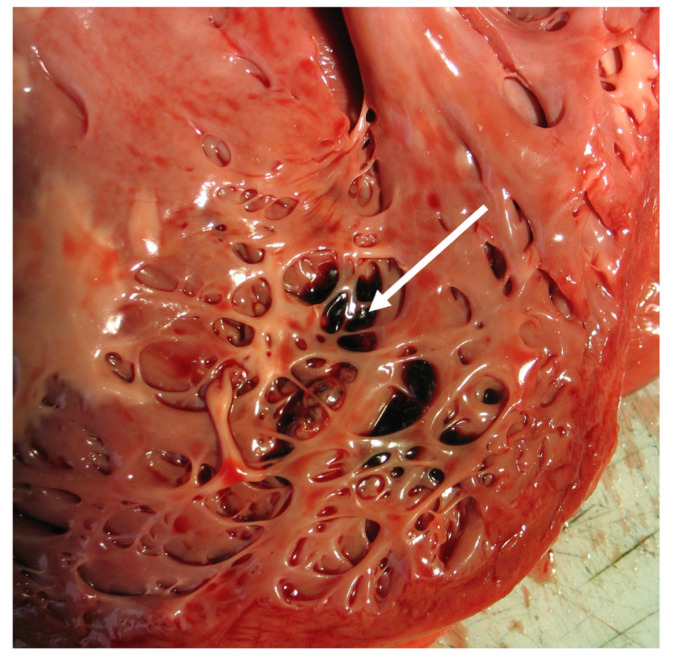
Autopsy specimen: left ventricle of a patient with NCCM and sudden cardiac death. A thin compacted layer and extensive trabeculation in the apical region. Small thrombi between the trabeculations (arrows).

Pathoanatomical studies of NCCM revealed a marked trabecular meshwork with many intertrabecular recesses in the involved mural segments of the LV myocardium (Figure 5) [8,43,122]. The intertrabecular recesses are lined with endothelium [9], ending blindly in the external compact layer without a connection to the coronary circulation [9,122]. In autopsy studies, prominent trabeculations were found in the LV in up to 70% of a group of subjects without apparent clinical heart disease [123]. However, more than three trabeculations were found in only 4% of the patients. On basis of these data, Stöllberger et al. defined pathological LV trabeculations when more than three trabeculations apical to the papillary muscles were present on echocardiography [124].

Burke examined hearts in cases with NCCM by autopsies and found poorly formed papillary muscles in the LV, a distinct noncompacted zone in the LV and, often, in the right ventricle [125]. The patterns of the noncompacted area include anastomosing trabeculations and a polypoid endocardial surface. None of the pathological or histological findings was typical for either the isolated or nonisolated form of NCCM. Different cardiac abnormalities were seen in the nonisolated form, including epicardial coronary malformation, histiocytoid cardiomyopathy, ventricular septal defects, and conotruncal diseases [125]. Jenni reported scar tissue within the trabeculations and in the subendocardial area but not in the epicardial zone [78].

In histological examinations the trabeculations were covered with excessive fibrous tissue and elastin deposits, perhaps suggestive of some degree of subendocardial ischemia [115,125]. Oechslin et al. found an increased number of normally formed trabeculations in hypertrabeculation, while the histological appearance of NCCM was “far beyond being normal” [41]. Burke found no difference between the hearts of isolated and nonisolated noncompaction cardiomyopathy [125].

Ultrastructural investigations could give additional impact on the discussion of pathogenesis of noncompaction areas. Ultrastructural investigations of hearts with noncompaction/hypertrabeculation demonstrated alterations in the shape and number of mitochondria, sarcomeric alterations, and other morphological abnormalities such as lipid-like inclusions and enlarged interstitial spaces [126]. The findings were generally nonspecific. The reported abnormalities were most prominent in patients with neuromuscular disorders. The changes included elongated mitochondria, swollen mitochondria, and disruption of the usual parallel orientation between mitochondria and sarcomeres. Other myocardial diseases such as myocardial ischemia and hibernation have been reported to involve abnormalities in mitochondria equally [127].

## 11. Genetics in Noncompaction Cardiomyopathy

### 11.1. Basic Aspects

Familial cumulation of NCCM assumes a genetic background. Basic research showed that trabeculation is regulated by genes and that mutations in the NOTCH pathway regulator MIB1 cause noncompacted myocardium [118].

The genetic pathogenesis of NCCM is heterogeneous. In a majority of the adult patients with noncompaction cardiomyopathy, it is an autosomal dominant disorder. X-linked disorders, autosomal recessive, and mitochondrial (maternal) inheritance have also been described. The first genetic cause of isolated NCCM was initially described by Bleyl et al., when they identified mutations in the X-linked G4.5-Gen encoding for Tafazzin, the gene also responsible for Barth syndrome [128]. Affected children show cardiomyopathies, half of them with noncompaction, neutropenia and myopathy. Emery–Dreifuss muscular dystrophy is caused by a G4.5 mutation as well.

Gene mutations have been identified, that cause congenital heart disease with noncompaction; in patients with hypoplastic left heart syndrome and noncompaction a DTNA (α- dystrobrevin) mutation was identified. Dystrophin mutations are also involved in boys with Duchenne and Becker muscular dystrophies [129]. Whereas mutations in Nkx-2.5 mutations were reported in children with noncompaction, atrial septal defect and β-myosin heavy chain (MYH7) in patients with noncompaction and Ebstein anomaly [129]. Chromosomal abnormalities and syndromic patients have also been identified with noncompacted myocardium such as Coffin-Lowry syndrome, Sotos syndrome, Hunter–McAlpine syndrome, and Charcot–Marie–Tooth disease [130].

NCCM is often familial with an autosomal dominant inheritance but with variable penetrance and a high intrafamilial variability [131]. Studies in the 1980s and 1990s led to the discoveries that the sarcomere mutations cause cardiomyopathies. Mutations of genes, that are responsible for hypertrophic or dilated cardiomyopathy, were found in patients with NCCM as well [132]. Even primary restrictive cardiomyopathy shares the same sarcomeric genetic background [133]. Recent publications showed that nearly half of the affected genes in patients with NCCM were sarcomere genes relevant for the structure of contractile and non-contractile elements with single missense mutations [134]. MYH7 was involved in 48% of the sarcomere gene mutations. MYH7 and ACTC1 mutations had significant lower risk for MACE than MYBPC3 and TTN mutations [28]. Arrhythmic genes, non-sarcomere/non-arrhythmic genes, X-linked genes, genes associated with congenital heart disease, mitochondrial dysfunction genes and complex genotypes were found as well but in small numbers [28]. In some families with autosomal dominant NCCM associated with congenital heart disease (CHD), affected members may have very minor forms of CHD that may have normalized spontaneously, whereas other family members may have severe forms of CHD. In addition, mutations in the sodium channel gene SCN5A, were reported to cause noncompacted myocardium and rhythm disturbance [135]. Genetic testing in patients with NCCM appears to detect clinically significant variants in 35% to 40% of tested individuals. Table 7 shows a selection of affected genes.

Children more frequently had an X-linked or mitochondrial inherited defect or chromosomal anomalies. In multivariate analysis MYBPC3, TTN, arrhythmia—non-sarcomere non-arrhythmia cardiomyopathy—and X-linked genes were genetic predictors for MACE. The presence of pathogenic variants was an independent risk factor for adverse outcomes in other cohorts as well and may aid in risk stratification in patients. Biallelic mutations and double pathogenic variants were found to have a worse prognosis [136,137].

Current investigations in more than 800 patients showed a genetic overlap indicating that NCCM often represents a phenotypic variation of DCM or HCM, but also variants uniquely associated with NCCM [138].

### 11.2. Genetic Testing in Familial Noncompaction Cardiomyopathy

Up to 40% of NCCM cases may be familial, so family screening is recommended when the diagnosis of NCCM is assessed in a child [12]. NCCM is a heterogeneous condition, and genetic stratification plays a role in clinical management. Distinguishing genetic from nongenetic noncompaction should help to predict an outcome and to find adequate management and follow-up decisions tailored to genetic status [137]. When a definite diagnosis of NCCM is assessed, the diagnostic process should include genetic testing which will provide a relatively high probability to find sarcomeric mutations [28]. No pathogenetic variants were identified in patients with isolated LVNC in the absence of cardiac dysfunction or syndromic features. Consequently, the diagnostic yield of genetic testing in adult index patients with LVNC is low. Genetic testing is most beneficial in LVNC associated with other cardiac and syndromic features, in which it can facilitate the correct diagnosis, and is least useful in adults with isolated LVNC without a family history of noncompaction [139,140].

HRS/EHRA in 2011 recommended mutation specific testing of family members and appropriate relatives following the identification of a NCCM causative mutation in the index case (Class I). NCCM genetic testing can be useful (Class II a) for patients in whom a cardiologist has established a clinical diagnosis of NCCM, based on an examination of the patient’s clinical history, family history, and electrocardiographic/echocardiographic phenotype [141]. The German position paper for “Gendiagnostik bei kardiovaskulären Erkrankungen” in 2015 conferred genetic testing in a patient with an established clinical diagnosis of NCCM a Class IIA recommendation. The recommendations include a mutation-specific test in family members after identification of the causative mutation in the index case (Class I) [142]. Sensitivity for a genetic test at that time was 20–30%. [142]. The AHA in 2020 recommends a family history for ≥3 generations and clinical screening for cardiomyopathy in asymptomatic first-degree relatives. Genetic testing should be considered for the most clearly affected person in a family to facilitate family screening and management. For NCCM the use of the gene panel for the cardiomyopathy identified in association with the NCCM phenotype is proposed, following the data of Hershberger [140,143].

In the pediatric population, genetic testing should be considered in individuals with cardiomyopathy co-occurring with NCCM. The actual database does not suggest an indication for cardiomyopathy gene panel testing in individuals with isolated noncompaction in the absence of a family history of cardiomyopathy phenotype with dilatation or hypertrophy [93].

## 12. Prognosis

With no underlying common diagnostic criteria, the comparison of different cohorts is difficult. The prognosis of the patient populations with NCCM is dependent on the occurrence of heart failure, death and on the need for heart transplantation. Systolic function is an important risk factor; heart failure with a reduced ejection fraction (HFrEF) with an LV ejection fraction below 35% has a worse prognosis. Several MR imaging studies demonstrated a good prognosis in preserved LV function [13,45,144,145].

Long term survival of patients with isolated apical noncompaction and preserved ejection fraction was shown to be comparable with the general population [16]. The end diastolic diameter of the LV assessed by echocardiography and heart failure symptoms have prognostic impact as well. No major cardiovascular events occurred in the non-symptom-based group, whereas 15/48 (31%) symptomatically diagnosed patients experienced cardiovascular death or heart transplantation. Independent predictors of cardiovascular death or heart transplantation were heart failure patient graded NYHA III-IV, sustained ventricular arrhythmias and left atrial size [146]. Left bundle branch block, atrial fibrillation and neuromuscular comorbidities have also been identified as risk factors [51,62,147]

Murphy et al. found that 62% of the patients developed heart failure symptoms. The death rate was found to be only 2% in a follow-up time of up to 15 years with regular visits [40]. In children, Pignatelli et al. described a mortality rate of 14% in 3 years, but transient recovery as well [148]. The Australian childhood cardiomyopathy registry has found the prognosis in children usually present with predominant noncompaction phenotype to be worse than the prognosis for matched children with DCM. In this registry, the freedom from death or heart transplantation at 10 years was 48% and at 15 years 45% [32]. The data of the RICARDA study in contrast show a better prognosis in those with hypertrabeculation, long term results are still lacking [149]. Whether the prognosis of NCCM differs from the prognosis of DCM in adults is unclear. A multicenter study from the Netherlands showed no difference between the groups, while the Heidelberg group showed a better prognosis in DCM compared to NCCM [150,151]. Aung et al. reported that LVNC patients had a similar risk of cardiovascular mortality compared with a DCM control group. The incidence rates of all-cause mortality, stroke and systemic emboli, heart failure admission, cardiac transplantation, ventricular arrhythmias, and cardiac device implantation were 2.16, 1.54, 3.53, 1.24, 2.17, and 2.66, respectively per 100 person-years. Meta-regression and subgroup analyses of these data revealed that LV ejection fraction, and not the extent of LV trabeculation, showed an important influence on the variability of incidence rates [152]. CMR studies compared the outcome of adults with NCCM compared to DCM patients and found no difference in the prognosis [14,15,153].

Genetic testing also has an impact on the prognostic stratification (see genetics).

Romano et al. demonstrated global longitudinal strain (GLS) derived with CMR to be a prognostic factor even in NCCM [154]. The existence of late enhancement in MR imaging was found to be an additional risk factor [155,156]. Vaidya et al. examined a study group with isolated apical noncompaction versus a patient group of mid basal noncompaction localization and found a lower risk of all-cause mortality compared to the mid basal noncompaction localization even in groups with comparable cardiovascular risk factors. However, in general, patients with isolated apical NCCM showed a higher LV ejection fraction and were more frequently asymptomatic than those with mid basal noncompaction localization [16]. Additionally, a correlation of 5 years mortality to the number of affected segments was found [137]. Vaidya et al. compared patients with and without left atrial dilatation, defined as LAVI > 34 cm^2^/m^2^. Left atrial dilatation was present in half of the patients. Among the patients with left atrial dilatation 25% died, compared to 8% without left atrial dilatation. However, the patients with left atrial dilatation were significantly older, showing a greater frequency of hypertension, congestive heart failure, and atrial fibrillation [16]. Regression of noncompacted areas was associated with an improvement in LV systolic function and might be associated with a favorable prognosis in these patients [157].

## 13. Therapy

There is no specific therapy for NCCM today. Therapy has to address the clinical symptoms and to cover prognostic aspects. Heart failure therapy in patients with NCCM and reduced LV systolic function can be applied according to the heart failure guidelines [62]. Cardiac resynchronization therapy results in an improvement of LV function in patients with left bundle branch block [158]. The implantation of an LV assist device is documented in several case reports [159]. In a single heart transplantation center, NCCM was a rare cause for transplantation with 2% of the cohort [160].

Antiarrhythmic therapy depends on the clinical situation, the use of ablation therapy in supraventricular and ventricular tachycardia, pacemaker and ICD systems have been reported [48,161,162]. Ablation of an accessory pathway in WPW-syndrome in children could be successfully performed in 83% with improvement of a reduced LV function in three of four of those with reduced LV function [49]. Therapy of atrial fibrillation in patients with cardiomyopathies is challenging [163].

The 2008 ACC/AHA guidelines graded ICD implantation a Class II b indication in NCCM independent of the systolic LV function [164]. The current guidelines on the prevention of sudden cardiac death do not mention NCCM (AHA), or state that there are only few data that LV noncompaction by itself is an indication for an ICD implantation (ESC) [165,166].

Anticoagulation with VKA is recommended in cases with NCCM and reduced ejection fraction with LVEF < 40%, as proposed in the literature [40,41,62]. The patients with NCCM after thromboembolic events and those with atrial fibrillation should also receive anticoagulation therapy. In patients with atrial fibrillation NOAC can be used instead of VKA as well.

Recently, there were case reports on resection of the noncompacted myocardium that showed recovery of the cardiac function. Long term follow-up is recommended [167].

**Figure 6:**
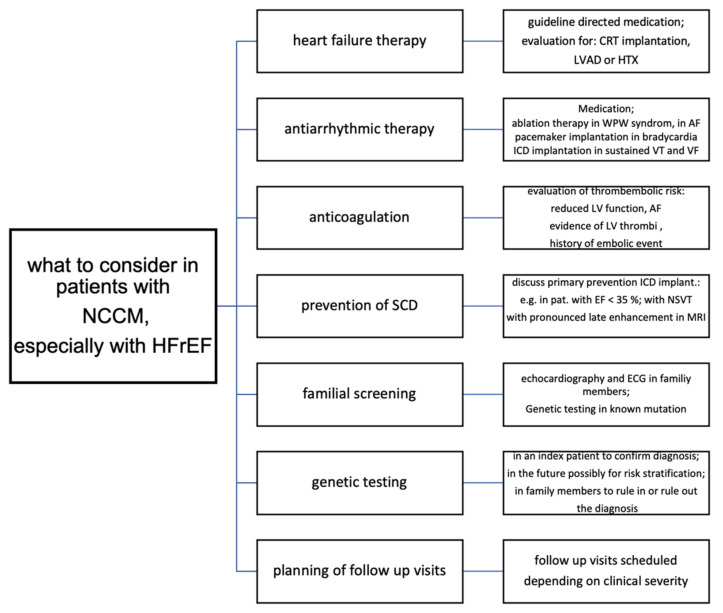
Treatment algorithm for patients with NCCM.

Regular physical activity, including systematic exercise, is an important component of prevention and therapy for most cardiovascular diseases and is associated with reduced mortality [168]. The 2020 ESC Guideline on sports cardiology regard NCCM patients and allow participation in high-intensity exercise and competitive sports only in asymptomatic individuals with an LVEF > 50% and the absence of arrhythmias, and in recreational exercise programs only in patients with an LVEF > 40%. Follow-up visits are recommended [168]. Figure 6 shows a proposal for a clinical algorithm for the management of noncompaction cardiomyopathy.

## 14. Future Work

Today, we know that the morphological noncompaction pattern is not restricted to the genetically determined NCCM. LVNC can occur in both, physiologic and pathologic remodeling. However, the same is true for other structural features such as dilatation of the LV or for LV hypertrophy, which can also be detected in a variety of clinical settings and require a differentiated approach.

Nevertheless, we need diagnostic consensus criteria to streamline future research efforts and be able to better compare patients’ subgroups with statistical meaningful volume. A big international registry could be the basis for more evidence-based recommendations to avoid unnecessary diagnostic testing and to formulate specific treatment options for this elusive cardiomyopathy.

There are ample areas of need for future research to help unravel the mysteries of this rare disease, NCCM. Future basic research should investigate the role of genetics as well as the ultrastructural features of cardiomyocytes to help discover targeted treatments. Future clinical studies need to focus on improving diagnostic imaging and laboratory testing. In the meantime, patients with NCCM should receive early diagnosis, counselling and optimal treatment, while avoiding overdiagnosis and overtreatment in those with only a physiologic remodeling.

## Figures and Tables

**Table 1:** A selection of terms and abbreviations for noncompacted cardiomyopathy and the noncompacted phenotype.

Term	Abbreviation
Spongy myocardium	
Fetal myocardium	
Honeycomb myocardium	
Persistent sinusoids	
Isolated noncompaction of the left ventricular myocardium	INVM
Left ventricular noncompaction	LVNC
Noncompaction cardiomyopathy	NCCM
Left ventricular hypertrabeculation	LVHT
Hypertrabeculation syndrome	
Left ventricular myocardial noncompaction cardiomyopathy	
Non-compaction of the left ventricular myocardium	

**Table 2:** Subtypes of noncompaction cardiomyopathies.

**(a) Subtypes of NCCM in the Pediatric Population,** modified after [12]
1. The isolated or benign form of LVNC, (M _LVNC_);
2. The arrhythmogenic form of LVNC;
3. The dilated form of LVNC, (M _LVNC + D_);
4. The hypertrophic form of LVNC, (M _LVNC + H_);
5. The “mixed” form of LVNC;
6. The restrictive form of LVNC, (M _LVNC + R_);
7. The biventricular form of LVNC;
8. The right ventricular hypertrabeculation with normal LV form;
9. The congenital heart disease form of LVNC.
**(b) Subtypes of NCCM,** modified after [27]
1. iLVNC. NC morphology in left ventricles with normal systolic and diastolic function, size, and wall thickness;
2. LVNC with LV dilation and dysfunction at onset, such as in the paradigmatic infantile CMP of Barth syndrome;
3. LVNC in hearts fulfilling the diagnostic criteria for DCM, HCM, RCM, or ARVC;
4. LVNC associated with congenital heart disease;
5. Syndromes with LVNC, either sporadic or familial, in which the noncompaction morphology is one of the cardiac traits associated with both monogenic defects and chromosomal anomalies, i.e., complex syndromes with several multiorgan defects;
6. Acquired and potentially reversible LVNC, which has been reported in athletes; it has also been reported in sickle cell anemia, pregnancy, myopathies, and chronic renal failure;
7. Right ventricular noncompaction, concomitant with that of the left ventricle, or present as a unique anatomic area of NC.

**Table 3:** Observed arrhythmias in patients with NCCM.

Type of Arrhythmia	Subtype	Prevalence
Bradyarrhythmias	Sinus bradycardia	
	First-degree AV block	
	Second-degree AV block	
	Mobitz II	
	Third-degree AV block	
	indication for	
	pacemaker implantation	5%
Supraventricular	Atrial fibrillation	18%
Tachycardias	Atrial flutter	1.5%
	Atrial tachycardia	
	AV nodal reentrant tachycardia	1.0%
	AV reentrant tachycardia	1.5%
Ventricular arrhythmias	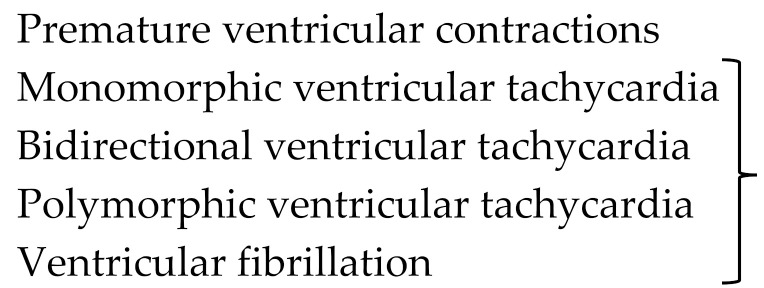	6%

AV—atrioventricular. Data from the German NCCM registry [47,48].

**Table 4:** Echocardiographic criteria for the diagnosis of NCCM in the German NCCM registry [62].

1. At least four prominent trabeculations and deep intertrabecular recesses;
2. Blood flow between the cavity of the left ventricle and the recesses demonstrable by color Doppler echocardiography or by the use of ultrasonographic contrast medium;
3. The left ventricular wall segments show a typical bilaminar structure, and the noncompact subendocardial layer is at least twice as thick as the compact subepicardial layer in systole;
4. No other cardiac abnormalities present.

**Table 5:** Different echocardiographic criteria for the diagnosis of NCCM.

Author Year; [ref.]	Appellation	Used Criteria						Cardiac Phase Used for Measurement	Recommended Views
		Trabeculations	Intertrabecular Recesses	Two-Layered Myocardial Structure	NC/C Ratio	Coexisting Cardiac Disease	Additional Criteria		
Chin 1990; [9]	California	Excessive prominent	Deep intertrabecular		X/Y ratio decrease i.e., C/NC+C; no exact cut-off value	Abnormalities excluded		End diastole	Apical view; subcostal view
Jenni 2001; [8,78]	Zurich	Excessive prominent trabeculations	Deep intertrabecular recesses	Compacted thin epicardial and much thicker noncompacted endocardial	NC/C > 2	Abnormalities absent	Perfused recesses in color Doppler	End systole	Short axis view
Stöllberger 2004; [17]	Vienna	>3 prominent trabeculations	Intertrabecular spaces	Trabeculations as part of noncompacted layer	No exact cut-off value		Perfusion of intertrabecular spaces by color Doppler	Trabeculations in end diastole; two-layered myocardium in end systole	Parasternal short axis and apical level; atypical apical 2-Ch view
Engberding 2007; [62]	Germany	At least 4 prominent trabeculations	Deep intertrabecular recesses	Bilaminar structure	NC/C ≥ 2	No other cardiac abnormalities	Blood flow in recesses in color Doppler or with echo contrast	Systole	
Belanger 2008; [72]	New York	Trabeculations	Recesses		NC/C	Absence of cardiomyopathy, congenital HD or coronary HD	Planimetered area of noncompacted myocardium	Systole	All standard views
Paterick 2012; [70]	Milwaukee	Trabeculations			NC/C > 2		Abnormal ventricular function	Total cardiac cycle; NC/C ratio end diastole	Multiple imaging windows
Van Dalen 2008; [75]	Rotterdam						Absence of LV twist		
Gebhard 2012; [73]	Additional						Compacta thickness < 8 mm		

**Table 6:** Different CMR criteria for the diagnosis of NCCM.

Author Year; [ref.]	Used Criteria					Cardiac Phase Used for Measurement	Recommended Views
	Trabeculations	Recesses	Two-layered structure	NC/C ratio	Additional criteria		
Petersen 2005; [85]	Trabecular layering		Compacted epicardial and noncompacted endocardial layer	NC/C > 2.3	True apex excluded	End diastole	Long axis
Jacquier 2010; [87]	Trabeculated LV mass	Perfused, deep recesses	Jenni echo criteria		Trabeculated mass > 20%	End diastole	Short axis
Stacey 2012; [86]	Trabeculation	Flow in the recesses	Noncompacted and compacted layer	NC/C > 2.0	16–24 mm from the true apex	End systole	Short axis
Captur 2015; [88]	Abnormal trabecular pattern		Jenni echo criteria and #		Maximum apical fractal dimension > 1.3; global fractal dimension > 1.26	End diastole	Short axis
Grothoff 2012; [89]	Trabeculations	Recesses communicating with the left ventricular cavity	Noncompacted/compacted myocardium ratio	NC/C > 2 (segments 4–6) NC/C > 3 (segments 1–3, 7–16) *	Trabeculated mass > 25% of total LV mass; Trabeculated LV mass/BSA > 15 g/m^2^	End diastole	Short axis
Choi 2016; [92]	Trabeculated mass		Most prominent noncompacted to compacted ratio	NC/C > 3.15 apical	Trabeculated mass > 35% of total LV volume	End diastole	Short axis

# One of the following: family history, neuromuscular disorders, regional wall motion abnormality, arrhythmia, heart failure, thromboembolic event. * According to the 17-segments model of the left ventricle.

**Table 7:** Genes involved in different forms of noncompaction cardiomyopathy.

Genes	Mutations in Gen:
Sarcomere genes	MYH7; MYBPC3; ACTC1; TNT;
(Contractile and non-contractile	TPM1; AN2; ACTN2; DES; LDB3;
Structures)	MYL2; NEBL; OBSCN; TNNC1; TNNI3
Arrhythmia genes	HCN4; RYR2; SCN5A; ABCC9;
	ANK2; CACNA2D1; CASQ2; KCNE3
	KCNH2; KCNQ1
Non-sarcomere/non-arrhythmia	MMPK; DSP; DTNA; FKTN; HFE; JUP;
Cardiomyopathy genes	LMNA (Lamin A/C); PKP2; PLEC; PLN;
	PRDM16; RBM20; SGCD
X-linked genes	G4.5 (TAZ); DMD; FHL1; GLA;
	LAMP2: RPS6KA3
Genes associated with	MIB1; MIB2; NKX2.5; NOTCH1;
congenital heart disease	NSD1; PTPN11; TXB20; TBX5
Mitochondrial dysfunction genes	HADHB; HMGCL; MIPEP; MLYCD
	MT-ATP6; MT-CO3; MTFMT; MT-ND1;
	MT-ND2; SDHA; SDHD; TMEM70; VARS2
Complex genotypes	Multiple mutations in one patient. Complex MYBPC3 mutations with severe clinical phenotype, observed only in children

Adapted from [28,134].
